# 1-Chloro-2-(4-phenyl­piperazin-1-yl)ethanone

**DOI:** 10.1107/S1600536809008216

**Published:** 2009-05-07

**Authors:** Yong-Ji Xu, Fei Jing

**Affiliations:** a College of Chemistry and Molecular Engineering, Qingdao University of Science and Technology, Qingdao 266042, People’s Republic of China

## Abstract

The title compound, C_12_H_15_ClN_2_O, is a piperazine derivative with the potential for use as a starting material for pharmaceutial and agrochemical applications. The structure is stabilized by C—H⋯O hydrogen bonds, C—H⋯π inter­actions and π–π stacking inter­actions [centroid–centroid distance = is 4.760 (2) Å].

## Related literature

For the biological activity of piperazine and its derivatives, see: Berkheij (2005 ▶); Upadhayaya *et al.* (2004 ▶); Choudhary *et al.* (2006 ▶); Vacca *et al.* (1994 ▶); Hulme (1999 ▶). For reference structural data, see: Drew & Leslie (1986 ▶).
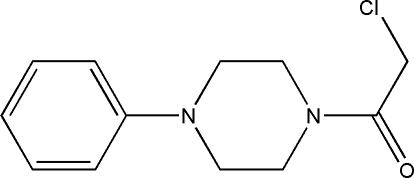


## Experimental

### 

#### Crystal data

C_12_H_15_ClN_2_O
*M* *_r_* = 238.71
Monoclinic, 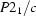

*a* = 9.4423 (19) Å
*b* = 8.5629 (17) Å
*c* = 14.506 (3) Å
β = 101.34 (3)°
*V* = 1149.9 (4) Å^3^
*Z* = 4
Mo *K*α radiation
μ = 0.31 mm^−1^
*T* = 113 K
0.20 × 0.18 × 0.12 mm

#### Data collection

Rigaku Saturn diffractometer
Absorption correction: multi-scan (*CrystalClear*; Rigaku, 2005 ▶) *T*_min_ = 0.940, *T*_max_ = 0.964
9264 measured reflections
2729 independent reflections
2151 reflections with *I* > 2σ(*I*)
*R*_int_ = 0.035

#### Refinement

*R*[*F* ^2^ > 2σ(*F* ^2^)] = 0.035
*wR*(*F* ^2^) = 0.098
*S* = 1.07
2729 reflections
145 parameters
H-atom parameters constrained
Δρ_max_ = 0.25 e Å^−3^
Δρ_min_ = −0.26 e Å^−3^

### 

Data collection: *CrystalClear* (Rigaku, 2005 ▶); cell refinement: *CrystalClear*; data reduction: *CrystalClear*; program(s) used to solve structure: *SHELXS97* (Sheldrick, 2008 ▶); program(s) used to refine structure: *SHELXL97* (Sheldrick, 2008 ▶); molecular graphics: *SHELXTL* (Sheldrick, 2008 ▶); software used to prepare material for publication: *SHELXTL*.

## Supplementary Material

Crystal structure: contains datablocks I, global. DOI: 10.1107/S1600536809008216/hg2461sup1.cif

Structure factors: contains datablocks I. DOI: 10.1107/S1600536809008216/hg2461Isup2.hkl

Additional supplementary materials:  crystallographic information; 3D view; checkCIF report

## Figures and Tables

**Table 1:** Hydrogen-bond geometry (Å, °)

*D*—H⋯*A*	*D*—H	H⋯*A*	*D*⋯*A*	*D*—H⋯*A*
C2—H2⋯O1^i^	0.93	2.47	3.1850 (16)	134
C9—H9*A*⋯O1	0.97	2.38	2.7456 (17)	102
C12—H12*B*⋯O1^ii^	0.97	2.43	3.3426 (16)	157
C5—H5⋯*Cg*2^iii^	0.93	3.25	3.7651 (15)	117
C8—H8*B*⋯*Cg*2^iv^	0.97	3.09	4.0393 (12)	168
C12—H12*A*⋯*Cg*2^iv^	0.97	3.04	3.6878	125

Symmetry codes: (i) 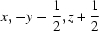; (ii) 
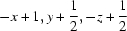
; (iii) 
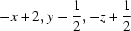
; (iv) 
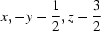
. *Cg*2 is the centroid of the C1–C6 ring.

**Fig. 1.**
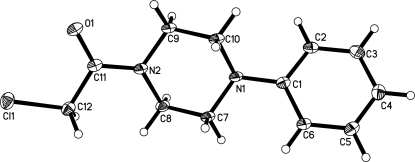
The molecular structure of (I), with atom labels and 30% probability displacement ellipsoids.

**Fig. 2.**
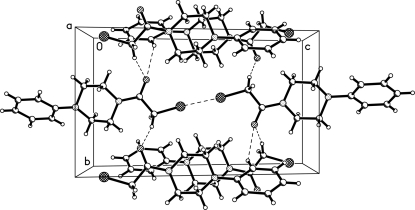
The packing diagram of (I), viewed down the c axis, showing the intermolecular hydrogen bonds (dashed lines).

## supplementary crystallographic information

### Comment

Piperazine and its derivatives are important pharmacores that can be found in biologically active compounds across a number of different therapeutic areas (Berkheij, 2005), such as antifungal (Upadhayaya *et al.*, 2004), anti-bacterial, anti-malarial, anti-psychotic agents (Choudhary *et al.*, 2006), HIV protease inhibitor (Vacca *et al.*, 1994), anti-depressant and anti-tumour activity colon, prostate, breast, lung and leukemia tumors (Hulme *et al.*, 1999). In an attempt to further synthesis piperazine derivatives, the title compound, 2-chloro-1-(4-phenylpiperazin-1-yl)ethanone, (I) (Fig. 1), was synthesized and its X-ray crystal structure determined.

In the  structure of title compund (Fig. 1), the bond lengths and angles in the piperazine ring and the benzene ring are normal (Drew & Leslie, 1986) (Table 1). The dihedral angle between the piperazine ring N1/N2/C7—C10 and C1—C6 benzene ring is 36.8 (2)°. The molecular structure is stabilized by inter and intramolecular C—H···O interactions (Table 2).  There exists π-π stacking interactions and C—H···π interactions. The π-π stacking interaction between the two phenyl rings is observed in the structure. The centroid distance between the two rings is 4.760 Å. There are three types of C—H···π interactions, C5—H5···C_g_2, C8—H8B···C_g_2 and C12—H12A···C_g_2 (C_g_2 is the C1—C6 ring centroid) (Table 2).

### Experimental

To a solution of 1-phenylpiperazine hydrochloride (2.0 g, 10 mmol) triethylamine (2.8 ml, 2 mmol) in anhydrous dichloromethane (50 ml) was added chloroacetyl chloride (0.8 mL, 10 mmol) dropwise at 273 K. The reaction mixture was stirred at room temperature for 2 h and monitored by TLC, and then the mixture was diluted with dichloromethane (50 ml) and washed with water (200 ml). The organic phase was dride over anhydrous sodium sulfate and concentrated to yield a solid which was crystallized to obtain 2-chloro-1-(4-phenylpiperazin-1-yl)ethanone.

### Refinement

H atoms were placed in calculated positions and treated using a riding model, with C—H = 0.93–0.98 Å and with *U*_iso_(H) = 1.2 *U*_eq_(C) or 1.5 *U*_eq_(C) for methyl H atoms.

### Crystal data

**Table:**

C_12_H_15_ClN_2_O	*F*(000) = 504
*M**_r_* = 238.71	*D*_x_ = 1.379 Mg m^−^^3^
Monoclinic, *P*2_1_/*c*	Mo *K*α radiation, λ = 0.71073 Å
Hall symbol: -P 2ybc	Cell parameters from 3260 reflections
*a* = 9.4423 (19) Å	θ = 3.2–27.9°
*b* = 8.5629 (17) Å	µ = 0.31 mm^−^^1^
*c* = 14.506 (3) Å	*T* = 113 K
β = 101.34 (3)°	Block, colourless
*V* = 1149.9 (4) Å^3^	0.20 × 0.18 × 0.12 mm
*Z* = 4	

### Data collection

**Table:**

Rigaku Saturn diffractometer	2729 independent reflections
Radiation source: rotating anode	2151 reflections with *I* > 2σ(*I*)
confocal	*R*_int_ = 0.035
ω scans	θ_max_ = 27.9°, θ_min_ = 3.2°
Absorption correction: multi-scan (*CrystalClear*; Rigaku, 2005)	*h* = −12→12
*T*_min_ = 0.940, *T*_max_ = 0.964	*k* = −8→11
9264 measured reflections	*l* = −16→19

### Refinement

**Table:**

Refinement on *F*^2^	Primary atom site location: structure-invariant direct methods
Least-squares matrix: full	Secondary atom site location: difference Fourier map
*R*[*F*^2^ > 2σ(*F*^2^)] = 0.035	Hydrogen site location: inferred from neighbouring sites
*wR*(*F*^2^) = 0.098	H-atom parameters constrained
*S* = 1.07	*w* = 1/[σ^2^(*F*_o_^2^) + (0.055*P*)^2^ + 0.0763*P*] where *P* = (*F*_o_^2^ + 2*F*_c_^2^)/3
2729 reflections	(Δ/σ)_max_ = 0.001
145 parameters	Δρ_max_ = 0.25 e Å^−^^3^
0 restraints	Δρ_min_ = −0.26 e Å^−^^3^

### Special details

**Table:**

Geometry. All e.s.d.'s (except the e.s.d. in the dihedral angle between two l.s. planes) are estimated using the full covariance matrix. The cell e.s.d.'s are taken into account individually in the estimation of e.s.d.'s in distances, angles and torsion angles; correlations between e.s.d.'s in cell parameters are only used when they are defined by crystal symmetry. An approximate (isotropic) treatment of cell e.s.d.'s is used for estimating e.s.d.'s involving l.s. planes.
Refinement. Refinement of *F*^2^ against ALL reflections. The weighted *R*-factor *wR* and goodness of fit *S* are based on *F*^2^, conventional *R*-factors *R* are based on *F*, with *F* set to zero for negative *F*^2^. The threshold expression of *F*^2^ > σ(*F*^2^) is used only for calculating *R*-factors(gt) *etc*. and is not relevant to the choice of reflections for refinement. *R*-factors based on *F*^2^ are statistically about twice as large as those based on *F*, and *R*- factors based on ALL data will be even larger.

### Fractional atomic coordinates and isotropic or equivalent isotropic displacement parameters (Å^2^)

**Table:**

	*x*	*y*	*z*	*U*_iso_*/*U*_eq_	
Cl1	0.62862 (4)	0.03256 (4)	0.08928 (2)	0.03631 (13)	
O1	0.58185 (10)	−0.16571 (10)	0.24044 (7)	0.0297 (2)	
N1	0.75320 (11)	0.03812 (12)	0.56187 (8)	0.0237 (2)	
N2	0.66086 (12)	−0.00754 (12)	0.36497 (8)	0.0244 (2)	
C1	0.82553 (13)	0.04592 (14)	0.65699 (10)	0.0230 (3)	
C2	0.76046 (14)	−0.02277 (14)	0.72590 (10)	0.0276 (3)	
H2	0.6724	−0.0739	0.7082	0.033*	
C3	0.82561 (16)	−0.01541 (15)	0.81981 (11)	0.0312 (3)	
H3	0.7815	−0.0627	0.8646	0.037*	
C4	0.95649 (16)	0.06196 (16)	0.84812 (10)	0.0311 (3)	
H4	1.0004	0.0664	0.9113	0.037*	
C5	1.02012 (14)	0.13211 (15)	0.78058 (10)	0.0299 (3)	
H5	1.1069	0.1854	0.7989	0.036*	
C6	0.95647 (13)	0.12419 (15)	0.68604 (10)	0.0253 (3)	
H6	1.0013	0.1714	0.6416	0.030*	
C7	0.80673 (14)	0.13837 (14)	0.49496 (10)	0.0256 (3)	
H7A	0.8961	0.0961	0.4821	0.031*	
H7B	0.8263	0.2419	0.5215	0.031*	
C8	0.69545 (14)	0.14856 (14)	0.40466 (10)	0.0266 (3)	
H8A	0.6084	0.1975	0.4170	0.032*	
H8B	0.7325	0.2128	0.3597	0.032*	
C9	0.61886 (13)	−0.11941 (14)	0.43097 (10)	0.0250 (3)	
H9A	0.6128	−0.2231	0.4035	0.030*	
H9B	0.5242	−0.0921	0.4425	0.030*	
C10	0.72706 (13)	−0.12050 (14)	0.52343 (10)	0.0256 (3)	
H10A	0.6908	−0.1857	0.5683	0.031*	
H10B	0.8174	−0.1650	0.5137	0.031*	
C11	0.63200 (13)	−0.03982 (14)	0.27233 (10)	0.0237 (3)	
C12	0.66787 (14)	0.08973 (15)	0.20873 (9)	0.0272 (3)	
H12A	0.7695	0.1158	0.2266	0.033*	
H12B	0.6124	0.1823	0.2169	0.033*	

### Atomic displacement parameters (Å^2^)

**Table:**

	*U*^11^	*U*^22^	*U*^33^	*U*^12^	*U*^13^	*U*^23^
Cl1	0.0485 (2)	0.0282 (2)	0.0289 (2)	0.00339 (14)	−0.00042 (16)	−0.00073 (13)
O1	0.0291 (5)	0.0214 (5)	0.0375 (6)	−0.0028 (4)	0.0034 (4)	−0.0060 (4)
N1	0.0219 (5)	0.0164 (5)	0.0314 (6)	−0.0042 (4)	0.0021 (4)	0.0009 (4)
N2	0.0241 (5)	0.0174 (5)	0.0309 (6)	−0.0036 (4)	0.0033 (4)	−0.0005 (4)
C1	0.0199 (6)	0.0161 (6)	0.0319 (7)	0.0025 (4)	0.0027 (5)	0.0008 (5)
C2	0.0236 (6)	0.0199 (6)	0.0396 (8)	−0.0006 (5)	0.0072 (6)	0.0022 (5)
C3	0.0358 (7)	0.0233 (7)	0.0360 (8)	0.0027 (6)	0.0105 (6)	0.0043 (6)
C4	0.0359 (7)	0.0263 (7)	0.0293 (8)	0.0046 (6)	0.0021 (6)	−0.0028 (6)
C5	0.0261 (7)	0.0254 (7)	0.0364 (8)	−0.0013 (5)	0.0016 (6)	−0.0042 (6)
C6	0.0222 (6)	0.0210 (6)	0.0325 (8)	−0.0023 (5)	0.0047 (5)	−0.0005 (5)
C7	0.0242 (6)	0.0181 (6)	0.0333 (8)	−0.0051 (5)	0.0028 (5)	0.0003 (5)
C8	0.0306 (7)	0.0164 (6)	0.0314 (7)	−0.0031 (5)	0.0026 (6)	−0.0005 (5)
C9	0.0228 (6)	0.0169 (6)	0.0348 (8)	−0.0049 (5)	0.0048 (5)	−0.0006 (5)
C10	0.0220 (6)	0.0160 (6)	0.0375 (8)	−0.0023 (5)	0.0031 (5)	0.0013 (5)
C11	0.0165 (6)	0.0193 (6)	0.0341 (7)	0.0031 (5)	0.0022 (5)	−0.0011 (5)
C12	0.0280 (7)	0.0220 (6)	0.0291 (7)	0.0016 (5)	−0.0004 (5)	−0.0014 (5)

### Geometric parameters (Å, °)

**Table:**

Cl1—C12	1.7682 (14)	C5—C6	1.386 (2)
O1—C11	1.2304 (15)	C5—H5	0.9300
N1—C1	1.4157 (18)	C6—H6	0.9300
N1—C7	1.4586 (16)	C7—C8	1.5119 (18)
N1—C10	1.4705 (16)	C7—H7A	0.9700
N2—C11	1.3463 (18)	C7—H7B	0.9700
N2—C9	1.4632 (17)	C8—H8A	0.9700
N2—C8	1.4666 (15)	C8—H8B	0.9700
C1—C6	1.3965 (18)	C9—C10	1.5177 (18)
C1—C2	1.4015 (19)	C9—H9A	0.9700
C2—C3	1.381 (2)	C9—H9B	0.9700
C2—H2	0.9300	C10—H10A	0.9700
C3—C4	1.391 (2)	C10—H10B	0.9700
C3—H3	0.9300	C11—C12	1.5229 (18)
C4—C5	1.383 (2)	C12—H12A	0.9700
C4—H4	0.9300	C12—H12B	0.9700
			
C1—N1—C7	117.19 (10)	H7A—C7—H7B	108.2
C1—N1—C10	115.21 (10)	N2—C8—C7	110.53 (10)
C7—N1—C10	110.21 (11)	N2—C8—H8A	109.5
C11—N2—C9	119.41 (10)	C7—C8—H8A	109.5
C11—N2—C8	124.38 (11)	N2—C8—H8B	109.5
C9—N2—C8	114.07 (11)	C7—C8—H8B	109.5
C6—C1—C2	118.20 (13)	H8A—C8—H8B	108.1
C6—C1—N1	123.06 (12)	N2—C9—C10	111.12 (10)
C2—C1—N1	118.70 (11)	N2—C9—H9A	109.4
C3—C2—C1	120.76 (13)	C10—C9—H9A	109.4
C3—C2—H2	119.6	N2—C9—H9B	109.4
C1—C2—H2	119.6	C10—C9—H9B	109.4
C2—C3—C4	120.68 (13)	H9A—C9—H9B	108.0
C2—C3—H3	119.7	N1—C10—C9	111.28 (10)
C4—C3—H3	119.7	N1—C10—H10A	109.4
C5—C4—C3	118.83 (14)	C9—C10—H10A	109.4
C5—C4—H4	120.6	N1—C10—H10B	109.4
C3—C4—H4	120.6	C9—C10—H10B	109.4
C4—C5—C6	121.01 (13)	H10A—C10—H10B	108.0
C4—C5—H5	119.5	O1—C11—N2	122.85 (12)
C6—C5—H5	119.5	O1—C11—C12	121.70 (12)
C5—C6—C1	120.52 (13)	N2—C11—C12	115.44 (10)
C5—C6—H6	119.7	C11—C12—Cl1	111.30 (9)
C1—C6—H6	119.7	C11—C12—H12A	109.4
N1—C7—C8	109.74 (11)	Cl1—C12—H12A	109.4
N1—C7—H7A	109.7	C11—C12—H12B	109.4
C8—C7—H7A	109.7	Cl1—C12—H12B	109.4
N1—C7—H7B	109.7	H12A—C12—H12B	108.0
C8—C7—H7B	109.7		
			
C7—N1—C1—C6	−10.51 (17)	C11—N2—C8—C7	−143.92 (12)
C10—N1—C1—C6	121.65 (13)	C9—N2—C8—C7	52.87 (14)
C7—N1—C1—C2	166.96 (11)	N1—C7—C8—N2	−57.54 (14)
C10—N1—C1—C2	−60.88 (15)	C11—N2—C9—C10	145.88 (11)
C6—C1—C2—C3	−1.11 (18)	C8—N2—C9—C10	−50.00 (14)
N1—C1—C2—C3	−178.71 (11)	C1—N1—C10—C9	166.07 (10)
C1—C2—C3—C4	0.7 (2)	C7—N1—C10—C9	−58.57 (13)
C2—C3—C4—C5	0.3 (2)	N2—C9—C10—N1	52.08 (14)
C3—C4—C5—C6	−0.9 (2)	C9—N2—C11—O1	−6.05 (18)
C4—C5—C6—C1	0.5 (2)	C8—N2—C11—O1	−168.43 (12)
C2—C1—C6—C5	0.48 (19)	C9—N2—C11—C12	174.87 (11)
N1—C1—C6—C5	177.96 (11)	C8—N2—C11—C12	12.49 (17)
C1—N1—C7—C8	−164.61 (10)	O1—C11—C12—Cl1	−0.20 (15)
C10—N1—C7—C8	61.01 (13)	N2—C11—C12—Cl1	178.89 (9)

### Hydrogen-bond geometry (Å, °)

**Table:**

*D*—H···*A*	*D*—H	H···*A*	*D*···*A*	*D*—H···*A*
C2—H2···O1^i^	0.93	2.47	3.1850 (16)	134
C9—H9A···O1	0.97	2.38	2.7456 (17)	102
C12—H12B···O1^ii^	0.97	2.43	3.3426 (16)	157
C5—H5···Cg2^iii^	0.93	3.25	3.7651 (15)	117
C8—H8B···Cg2^iv^	0.97	3.09	4.0393 (12)	168
C12—H12A···Cg2^iv^	0.97	3.04	3.6878	125

Symmetry codes: (i) *x*, −*y*−1/2, *z*+1/2;  (ii) −*x*+1, *y*+1/2, −*z*+1/2; (iii) −*x*+2, *y*−1/2, −*z*+1/2; (iv) *x*, −*y*−1/2, *z*−3/2.
